# Genomic organisation analysis of novel immunoglobulin-like transcripts in Atlantic salmon (*Salmo salar*) reveals a tightly clustered and multigene family

**DOI:** 10.1186/1471-2164-11-697

**Published:** 2010-12-09

**Authors:** Anders E Østergaard, Krzysztof P Lubieniecki, Samuel AM Martin, René JM Stet, William S Davidson, Christopher J Secombes

**Affiliations:** 1 Scottish Fish Immunology Research Centre, University of Aberdeen, Zoology Building, Tillydrone Avenue, Aberdeen AB24 2TZ, UK; 2 Department of Molecular Biology and Biochemistry, Simon Fraser University, Burnaby, British Columbia, Canada V5A 1S6; 3 Cell Biology & Immunology Group, Department of Animal Sciences, Wageningen University, P.O. Box 338, 6700 AH Wageningen, The Netherlands

## Abstract

**Background:**

Several novel immunoglobulin-like transcripts (NILTs) which have previously been identified in the salmonid species rainbow trout (*Oncorhynchus mykiss*) contain either one or two extracellular Ig domains of the V-type. NILTs also possess either an immunoreceptor tyrosine-based activating motif (ITAM) or immunoreceptor tyrosine-based inhibitory motifs (ITIMs) in the cytoplasmic region resulting in different signalling abilities. Here we report for the first time the genomic organisation and structure of the multigene family of NILTs in Atlantic salmon (*Salmo salar) *using a BAC sequencing approach.

**Results:**

We have identified six novel Atlantic salmon NILT genes (*Ssa-NILT1-6*), two pseudogenes (*Ssa-NILTp1 *and *Ssa-NILTp2*) and seven genes encoding putative transposable elements in one BAC covering more than 200 kbp. *Ssa*-NILT1, 2, 4, 5 and 6 contain one Ig domain, all having a CX_3_C motif, whereas *Ssa*-NILT3 contains two Ig domains, having a CX_6_C motif in Ig1 and a CX_7_C motif in Ig2. Atlantic salmon NILTs possess several ITIMs in the cytoplasmic region and the ITIM-bearing exons are in phase 0. A comparison of identity between the amino acid sequences of the CX_3_C Ig domains from NILTs varies from 77% to 96%. *Ssa-NILT1*, *2*, *3 *and *4 *were all confirmed to be expressed either by their presence in EST databases (*Ssa-NILT1*) or RT-PCR (*Ssa-NILT2*, *3*, and *4*) using cDNA as template. A survey of the repertoire of putative NILT genes in a single individual revealed three novel genes (*Ssa-NILT7-9*) represented by the Ig domain, which together with Ig domains from *Ssa-NILT1-6 *could be divided into different groups based on specific motifs.

**Conclusions:**

This report reveals a tightly clustered, multigene NILT family in Atlantic salmon. By screening a highly redundant Atlantic salmon BAC library we have identified and characterised the genomic organisation of six genes encoding NILT receptors. The genes show similar characteristics to NILTs previously identified in rainbow trout, having highly conserved cysteines in the Ig domain and several inhibitory signalling motifs in the cytoplasmic region. In a single individual three unique NILT Ig domain sequences were discovered at the genomic DNA level, which were divided into two different groups based on a four residue motif after the third cysteine. Our results from the BAC screening and analysis on the repertoire of NILT genes in a single individual indicates that many genes of this expanding Ig containing NILT family are still to be discovered in fish.

## Background

Disease control and health is central to the production of salmon in aquaculture and more knowledge of the immune system in fish might help prevent infectious disease outbreaks. Salmon inhabit a temperate environment and their adaptive immune system is not as rapid as in mammals. Therefore, they rely to a greater degree on the innate immune system to combat pathogens [1]. A wide range of activating and inhibitory receptors play a role in the innate immune system, of which many are expressed on neutrophils, macrophages and natural killer (NK) cells. These receptors recognise conserved pathogen associated molecular patterns (PAMPs) released from or found on the surface of pathogens [2], and result in the activation of responsive cells. Many of these cell-surface receptors contain immunoglobulin-like (Ig) domains [3] and several genes have been found in clusters such as the leukocyte receptor complex (LRC) [4] and the triggering receptor expressed on myeloid cells (TREM) cluster [5] in mammals. The LRC is a very gene dense region, spanning 1 Mb, which includes the killer cell Ig-like receptors (KIRs), leukocyte Ig-like receptors (LILRs) and the natural cytotoxicity receptor (NCR) NKp46 [6]. The TREM cluster on human chromosome 6 harbours genes such as TREM1 and 2, as well as the NCR named NKp44 [5,7]. TREM receptors are involved in the amplification and attenuation of the inflammatory response [8,9], while the NKp44 receptor activates NK cells [10]. All these receptors are type I transmembrane proteins characterised by the presence of a variable number of extra-cellular Ig domains of either the C2-type or the novel V-type [10,11]. The Ig domains are usually followed by a connecting peptide, a transmembrane region, and a cytoplasmic tail. Some inhibitory receptors have a long cytoplasmic region containing one or more immunoreceptor tyrosine-based inhibitory motifs (ITIMs) [12], which block NK cell-mediated cytotoxicity [13,14]. The cytoplasmic regions of activating receptors are short and associate with adaptor molecules such as DAP12, CD3ζ or FcεRIγ via a positively charged residue (arginine or lysine) in their transmembrane region. These adaptor proteins contain a negatively charged residue in their transmembrane region and an immunoreceptor tyrosine-based activating motif (ITAM) in their cytoplasmic region [15].

In several species of teleost fish, receptors belonging to the Ig super family (IgSF) have been reported. These include the novel immune-type receptors (NITRs) reported in Southern pufferfish [16], zebrafish [17], channel catfish [18], rainbow trout [19], Japanese flounder [20] and sea bass [21], and the novel immunoglobulin-like transcript (NILT) genes described in carp [22] and rainbow trout [23,24]. Most NITRs possess a V-type Ig domain and most of them also have a second Ig domain of the V/C2-type followed by a transmembrane and cytoplasmic region. The majority of NITRs contain an ITIM, whereas a few contain an ITAM instead.

NILT receptors have either one or two extracellular Ig domains, a connecting peptide, a transmembrane region, and a cytoplasmic region containing the signalling motifs, and are expressed mainly in lymphoid tissues [23,24]. Homology modelling has indicated that these receptors have an extra-cellular Ig domain structurally similar to the V-type Ig domain of human NKp44. In carp, the *NILT *genes were shown to belong to a multigene family and analysis of the NILT Ig-encoding sequences revealed extensive haplotypic and allelic polymorphism [22]. Several *NILT *genes have been characterized in rainbow trout [24] suggesting the multigene status of *NILT *in salmonids. These observations prompted us to investigate the genomic organization and structure of *NILT *genes in Atlantic salmon using a bacterial artificial chromosome (BAC) library. This paper describes the first study on *NILT *in Atlantic salmon and reveals the genomic organization of several *NILT *genes in a single BAC clone sequence. In addition, the analysis shows that these *NILT *genes are tightly clustered in Atlantic salmon, with Ig domains structurally related to the novel V-type Ig domain of NKp44 and containing inhibitory signalling motifs.

## Results

### Identification and gene organization of *NILTs *in Atlantic salmon

**Figure 1:**
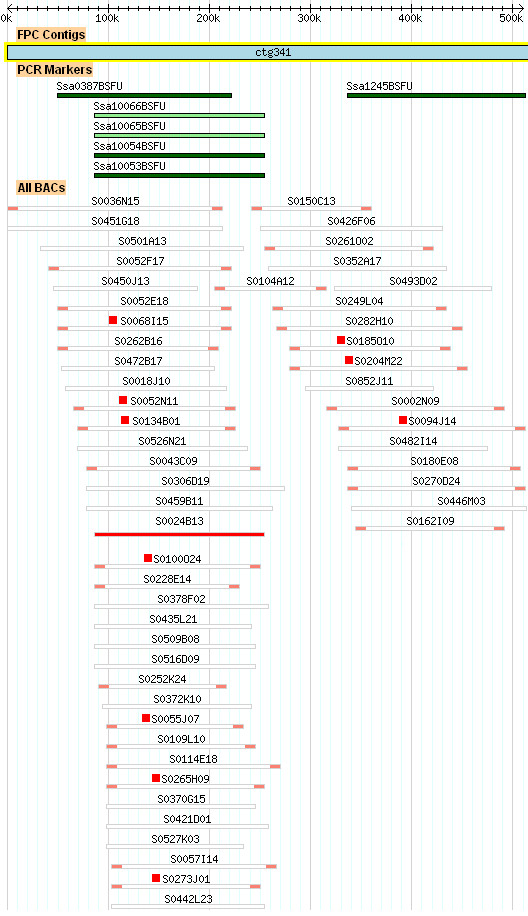
**Screenshot of FPC contig 341 adapted from http://www.asalbase.org**. BAC clone S0024B13 was chosen for shotgun sequencing (indicated in red). BAC clones positive for NILT hybridization probe are indicated by a red square. The position of PCR markers are highlighted with dark and light green bars. All BAC clones have been end sequenced (indicated in light red).

**Figure 2:**
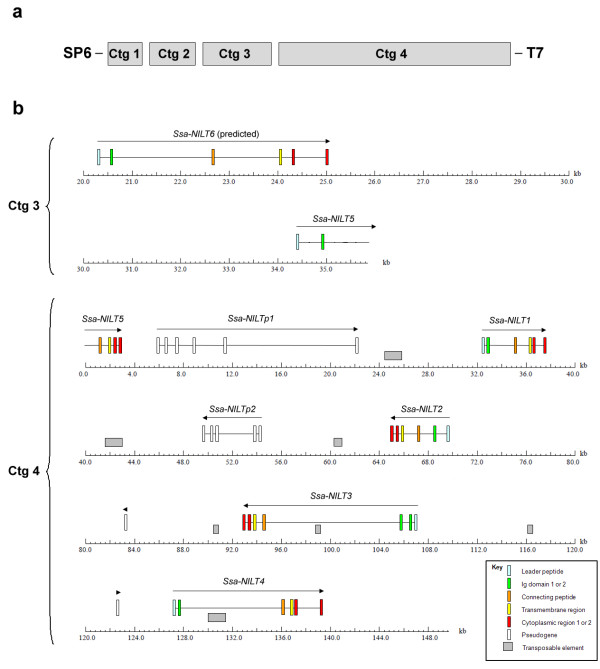
**Assembly of NILT-containing BAC clone**. a. Graphic illustration of the orientation and the number of contigs (ctg) after assembly. b. Genomic organization of the Atlantic salmon (*Ssa*) *NILT *genes identified in BAC clone S0024B13. Contig 3 contains a partial and a full-length predicted NILT. Contig 4 contains four full-length NILT genes, one partial NILT gene and two NILT pseudogenes.

**Figure 3:**
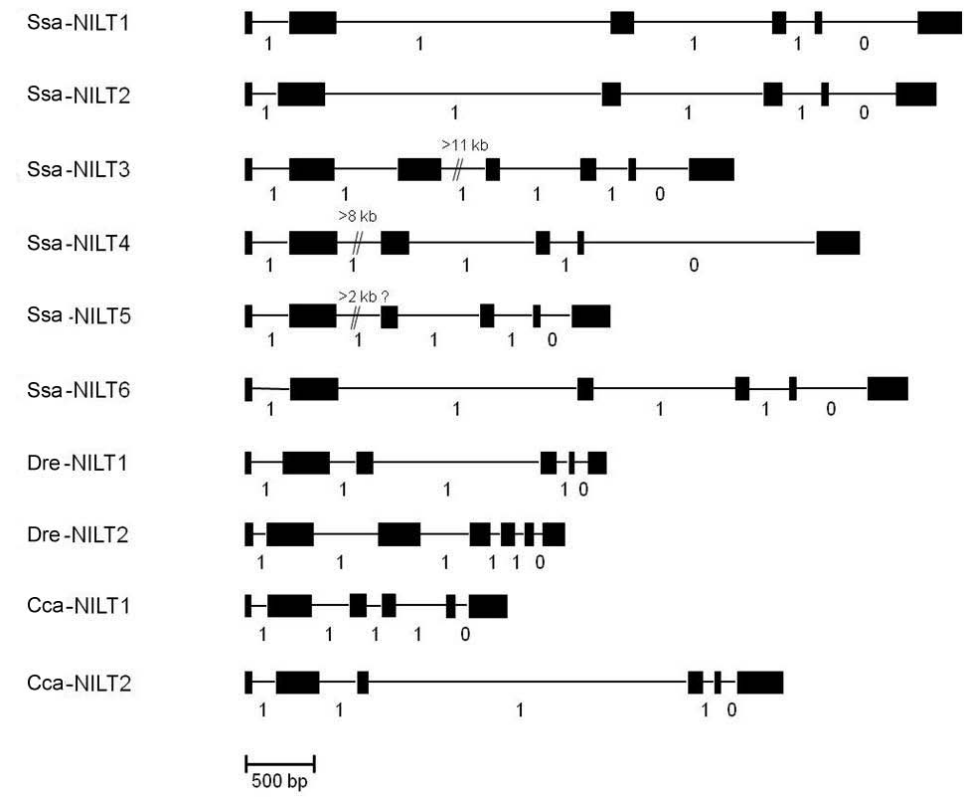
**Exon-intron structure of *NILT *genes from the Atlantic salmon (*Ssa*), zebrafish (*Dre*), and carp (*Cca*)**. The exons are indicated by boxes and introns by horizontal lines. Phase of the introns are shown below each intron.

Two partial NILT sequences [GenBank:DW550613, GenBank:DW564289] [25], from Atlantic salmon were used to design a 40-mer oligonucleotide probe located in the Ig domain. The probe hybridised to several BAC clones located within contig 341 in the physical map of the Atlantic salmon genome generated based on *Hin*dIII fingerprinting (Figure 1) [26], http://www.asalbase.org, of which one (S0024B13) was selected for shotgun sequencing. A continuous sequence of 149,704 bp was obtained after shotgun sequencing, contig assembling and gap closing using specific primers located in the contig ends [GenBank:GU552297]. Examination of this region (contig 4) identified four full-length *NILT *genes (*Ssa-NILT1*, *Ssa-NILT2*, *Ssa-NILT3 *and *Ssa-NILT4*) and one partial *NILT *gene (*Ssa-NILT5*). In the neighbouring contig 3 (size 35,964 bp) the putative 5'end of *Ssa-NILT5 *was identified as well as a predicted *Ssa-NILT6*. The NILT genes are orientated in both 3' and 5' directions in the BAC clone sequence (Figure 2). The open reading frames (ORF) of the genes containing one Ig domain are; *Ssa-NILT1 *(1032 bp), *Ssa-NILT2 *(993 bp), *Ssa-NILT4 *(1062 bp), *Ssa-NILT5 *(933 bp) and *Ssa-NILT6 *(945 bp) with each gene consisting of six exons and five introns. The Ig domain, connecting peptide and transmembrane region are encoded in exons 2, 3, and 4, respectively. Exon 4 also contains part of the cytoplasmic region, while the remainder of the cytoplasmic region and the 3' untranslated region are encoded in exons 5 (Cyt1) and 6 (Cyt2). Introns 1-4 are in phase 1, whereas intron 5 is in phase 0 (Figure 3). The *Ssa-NILT3 *(1278 bp) gene encodes the only NILT discovered so far in Atlantic salmon to have two Ig domains, and containing an additional intron in phase 1. The phases of the introns are highly conserved.

**Figure 4:**
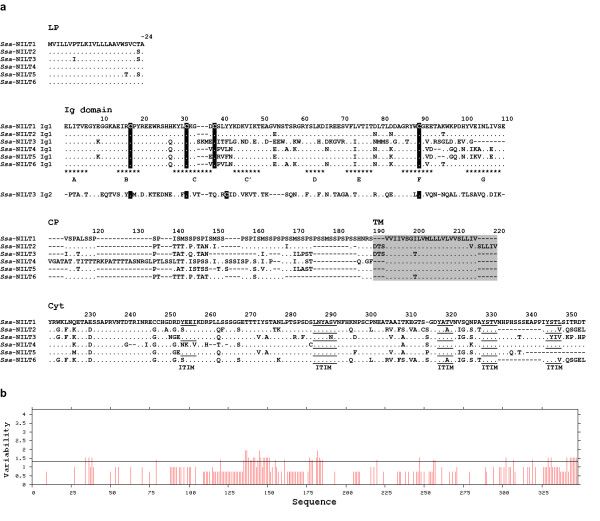
**Comparison and variability analysis of NILTs from Atlantic salmon**. a. Multiple sequence alignment of the deduced amino acid sequences of *Ssa*-NILT1-6 using Clustal W. Conserved cysteines are indicated in black. The transmembrane region is highlighted in gray. Putative inhibitory signalling motifs (ITIMs) in the cytoplasmic region are underlined. The predicted eight beta-strands are denoted by asterisks. LP, leader peptide; CP, connecting peptide; TM, transmembrane region; Cyt, cytoplasmic region. Dots (.) indicate identity to *Ssa*-NILT1 and dashes (-) denote gaps introduced for optimal alignment. b. Analysis of the variability of the entire Ssa-NILT protein sequences using Shannon entropy calculations. Positions with a Shannon entropy higher than 1.3 are considered significantly variable (indicated by black line).

The Ig domains of all the *Ssa*-NILTs are of the V-type and contain the characteristic cysteines at positions 17 and 89 responsible for disulphide bridge formation (Figure 4). Additional cysteines separated by three amino acids (CX_3_C) are present in the Ig domains of *Ssa*-NILT1, *Ssa*-NILT2, *Ssa*-NILT4, *Ssa*-NILT5 and *Ssa*-NILT6 whereas they are separated by six amino acids (CX_6_C) in the Ig1 domain of *Ssa*-NILT3. In the second Ig domain of *Ssa*-NILT3 they are separated by seven amino acids (CX_7_C). These cysteines may form a second disulphide bridge. The connecting peptide of *Ssa*-NILT1 contains a high number of serine/threonine residues, 29/56 respectively. Twenty-three serine residues were identified using the NetPhos server 2.0 http://www.cbs.dtu.dk/services/NetPhos/ in the connecting peptide of *Ssa*-NILT1 to be potential phophorylation sites (data not shown). This high proportion of serine and threonine residues is also observed in the connecting peptide of *Ssa*-NILT2-6 with 22/45, 15/33, 36/68, 22/38 and 20/37 residues, respectively (Figure 4). In addition, the Shannon entropy (*H*; http://imed.med.ucm.es/PVS/) was calculated for the *Ssa*-NILT sequences. Positions with *H *values above 1.3 are considered to be variable, while those with *H *values smaller than 1.3 are identified as conserved. The Shannon plot revealed that amino acid positions in the Ig domain and in the connecting peptide of the deduced NILT proteins could be considered as variable (Figure 4). The cytoplasmic regions of NILTs in Atlantic salmon range in size from 332 to 377 residues and the percentage of identity between the NILTs varies from 63% to 84% at the amino acid level. The NILTs have either four or five inhibitory motifs in the cytoplasmic region, which adhere to the consensus of ITIMs (S/I/V/LxYxxI/V/L or YxxI/V/L) (Figure 4). These correspond to the NILTs found in rainbow trout, which also contain several inhibitory signalling motifs [24]. *Ssa-NILT1*, *Ssa-NILT2*, *Ssa-NILT3*, and *Ssa-NILT4 *were all confirmed to be expressed either by their presence in EST databases http://web.uvic.ca/grasp (*Ssa-NILT1*) or RT-PCR (*Ssa-NILT2*, *Ssa-NILT3*, and *Ssa-NILT4*) using cDNA as template and primers listed in Table 1. We were not able amplify a specific product representing *Ssa-NILT5*, while a product for *Ssa-NILT6 *was amplified using genomic DNA as template. However, the primers used for amplifying a specific product representing *Ssa-NILT6 *were very difficult to design due to high similarity between NILT genes in the BAC sequence.

**Table 1:** Primer sequences for amplifying full-length Ssa-NILT2, 3 and 4, and Ig domains

Primers	Sequence (5'-3')	Amplifying
Ssa-NILT-f1	ACTAGCTGGGAGCCACAAGTCATC	*Ssa*-NILT2/*Ssa*-NILT4
Ssa-NILT-f2	CATCATGGTTATCTTGCTGGTCAT	*Ssa*-NILT3
Ssa-NILT-r1	TTGACGTTGGCGTAGTTGAG	*Ssa*-NILT2/*Ssa*-NILT3
Ssa-NILT-r2	GTCCCTTATGATTGGTTGTCAGTG	*Ssa*-NILT4
Ssa-panIg-f	GAGTTGATCACAGTGGAAGGA	Pan-NILT Ig
Ssa-panIg-r	TCCRCACCAGTATSTYCCAGC	Pan-NILT Ig

A pseudogene, *Ssa-NILTp1*, was identified between nucleotide position 6197 and 22738 in the BAC clone sequence (contig 4), comprising a leader peptide, two putative Ig domains, a connecting peptide, a transmembrane region and part of the cytoplasmic region (Figure 2). The pseudo gene contained a stop codon at nucleotide position 7013 in the Ig1 domain immediately after the last cysteine. Another pseudogene, *Ssa-NILTp2*, orientated in the reverse direction was identified between nucleotide position 49770 and 54621 in the same contig. *Ssa-NILTp2 *consists of a leader peptide and one Ig domain followed by the initial part of a second Ig domain, a transmembrane region and the cytoplasmic tail. An exon coding for the connecting peptide was not identified suggesting that it may not function as a true NILT. Two additional Ig domains, nucleotide position 83454 to 83746 and 122661 to 122780 were also discovered in contig 31, both of them containing either a stop codon or a nucleotide deletion interrupting the reading frame.

Intron 3 from *Ssa-NILT3 *is large (>11 kbp) and contained a conserved domain belonging to the reverse transcriptase (RT) superfamily. GENSCAN predictions [27] and BLASTn [28] searches of the non-redundant nucleotide database using the BAC clone sequence as query predicted seven transposable elements (Figure 2) two of which belong to the piggyBac-like DNA transposon family and one belongs to the Tc1-like transposon family [29]. The remaining four transposable elements contained conserved domains belonging to the RT superfamily.

**Figure 5:**
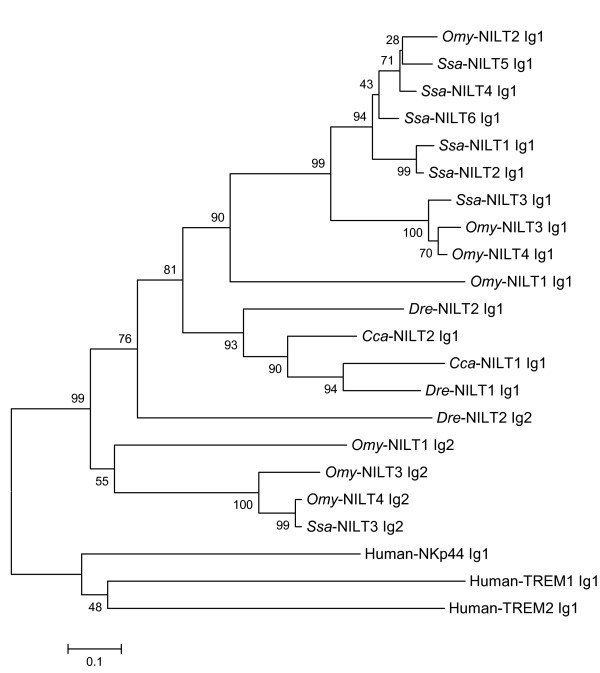
**Phylogenetic tree showing the relationship between NILTs from Atlantic salmon, rainbow trout, carp and zebrafish and NKp44 and TREM molecules from human using the amino acid sequences of the Ig domains**. The unrooted tree was built by the neighbour-joining method using Clustal W and the MEGA 4 packages and bootstrapped 10,000 times. The scale bar corresponds to 0.1 estimated amino acid substitutions per site. [GenBank: *Omy*-NILT1 [23]; *Omy*-NILT2, FM180056; *Omy*-NILT3, FM180057; *Omy*-NILT4, FM180058; *Cca*-NILT1, CAH19212; *Cca*-NILT2, CAH19213; *Dre*-NILT1, BN001234; *Dre*-NILT2, BN001235; Human-TREM1, AAL74018; Human-TREM2, AAH32362; Human-NKp44, AJ225109].

A phylogenetic tree was constructed using the amino acid sequence of the Ig domains of NILTs from Atlantic salmon, rainbow trout, carp and zebrafish together with human Ig receptors (Figure 5). It shows a clear division of Ig1 and Ig2 domains as well as clustering of salmonids and cyprinids.

### NILT repertoire in Atlantic salmon

Genomic DNA was extracted from a single individual and used as template in ten independent PCRs in order to amplify a specific part of the Ig domain. Ten clones from each PCR were sequenced and only NILT sequences observed more than once were included in the analysis of the repertoire. Eighty one clones were found positive for NILT resulting in seven different sequences and 19 singletons at the amino acid level. Despite using degenerate oligonucleotide primers designed to amplify Ig domains containing either CX_3_C or CX_6_C motifs (Table 1) only Ig domains containing CX_3_C motifs were observed.

**Figure 6:**
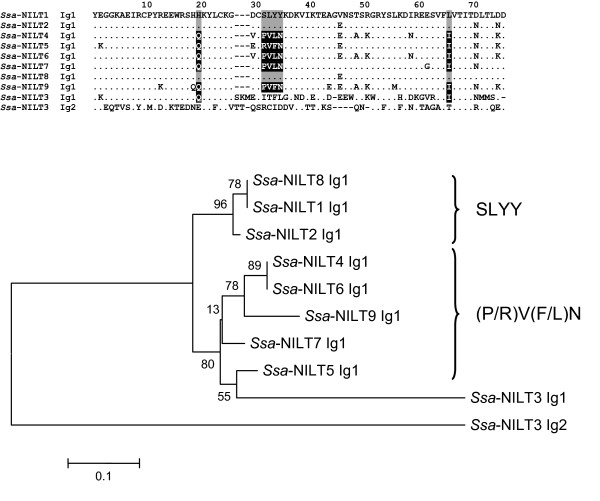
**Multiple alignment of the amino acid sequences of NILT Ig domains from the Atlantic salmon**. Characteristic residues are highlighted in black and gray. Dots (.) indicate identity to *Ssa*-NILT1 Ig1 and dashes (-) denote gaps introduced for optimal alignment. Below is a rooted phylogenetic tree showing the division into different groups of NILT Ig domains from the Atlantic salmon. The unrooted tree was built by the neighbour-joining method using Clustal W [41] and the MEGA 4 [42] packages and bootstrapped 10,000 times.

Of the seven different sequences retrieved, four were identical to either *Ssa-NILT1*, *Ssa-NILT2*, *Ssa-NILT4 *or *Ssa-NILT6 *at the nucleotide level, leaving three novel Ig domains of NILTs *Ssa-NILT7-9*. Multiple alignment of the amino acid sequences and subsequent phylogenetic analysis revealed a division of NILT Ig domains into two different types (Figure 6). Group 1, comprising *Ssa-NILT1*, *Ssa-NILT2*, and *Ssa-NILT8*, have Ig domains containing a CX_3_C motif as well as a histidine residue at position 20, a SLYY motif located at positions 32 to 35 (immediately after the third cysteine), and a leucine residue at position 66. Group 2, comprising *Ssa-NILT4*, *Ssa-NILT5*, *Ssa-NILT6*, *Ssa-NILT7*, and *Ssa-NILT9*, have Ig domains also containing a CX_3_C motif, but having a glutamine residue at position 20, a (P/R)V(F/L)N motif located from position 32 to 35, and an isoleucine residue at position 66. In the Ig1 domain of *Ssa-NILT3*, which contains a CX_6_C motif, there was a characteristic glutamine residue at position 20 and the isoleucine residue in position 66. The Ig2 domain, containing a CX_7_C motif, is significantly different and was included in the phylogenetic tree as an out-group.

## Discussion

Pattern recognition receptor (PRR) genes encoding the tertiary structure of an Ig domain belong to the immunoglobulin superfamily. These Ig containing receptors are found throughout the animal kingdom and several clusters have been identified in mammals [30]. A classic example is the leukocyte receptor complex, which contains several gene families encoding PRRs having single or multiple Ig domains [6]. It is hypothesised that they have evolved through duplication to give a wide array of Ig-containing PRRs within the innate immune system of vertebrates in order to recognise potential pathogenic organisms. In this paper we describe for the first time the characterisation and genomic organisation of the Ig-containing receptor termed novel immunoglobulin-like transcript (NILT) in the Atlantic salmon.

Six NILT genes, equally distributed within a BAC sequence (S0024B13), were identified. In addition, two pseudogenes as well as two single pseudo Ig domains and seven transposable elements were identified. Comparison of the genomic organisation revealed a similar structure for the NILT genes, with the exception of *Ssa-NILT3*, which contains an additional Ig domain (Ig2) encoded by exon 2a followed by a long intron 2a of 11 kbp (Figure 2). The occurrence of a NILT with two Ig domains was also present in rainbow trout [24], in which the intron following the Ig2 encoding exon in *Omy-NILT1 *has a size of 5.8 kb (Table 2) [23]. However, when examining the equivalent intron in *Dre-NILT1 *predicted from zebrafish, it was shown to be only 362 bp long [24] indicating a more compact gene structure of NILT genes in cyprinids compared to salmonids. Intron 1 in all Atlantic salmon NILTs, except for *Ssa-NILT2*, has the exact same size of 275 nucleotides and showed >96% identity. The remaining introns differ in length, however, intron 2 and 3 from *Ssa-NILT1 *and *Ssa-NILT2 *also revealed a high percentage of identity (>95%). Between different teleost species the sizes of introns and exons are generally highly conserved (Table 2), implying a close relationship of NILT genes across species. Comparison of the amino acid sequences of the Ig encoding exons from *Ssa-NILT1 *and *Ssa-NILT2 *revealed a strikingly high percentage of identity (96%) (Table 3). In contrast, the percent identity between the cytoplasmic region encoded by exon 5 and 6 of *Ssa-NILT1 *and *Ssa-NILT2 *was only 67%. The lowest percent of identity between Atlantic salmon Ig domains was seen when comparing Ig1 and Ig2 in *Ssa-NILT3 *(26%), which might indicate that NILT receptors with two Ig domains have a larger repertoire of binding specificities than NILT receptors with one Ig domain. So far no ligands for the NILT receptor have been identified, but future work will determine if there is any resemblance to the highly polymorphic KIRs in humans, which bind another highly polymorphic ligand, namely major histocompatibility complex (MHC) class I [31].

**Table 2:** Nucleotide lengths of exons and introns of NILTs found in teleost fish

	LP Exon 1	Intron 1	Ig1 Exon 2	Intron 2	Ig2 Exon 2a	Intron 2a	CP Exon 3	Intron 3	TM Exon 4	Intron 4	Cyt1 Exon 5	Intron 5	Cyt2 Exon 6	3'UTR	Signalling motif
***Ssa-NILT1***	46	275	348	2056	-	-	168	1074	96	218	50	753	324	395	5 ITIMs
***Ssa-NILT2***	46	180	348	2067	-	-	135	1063	120	286	50	511	294	2232	4 ITIMs
***Ssa-NILT3***	46	275	330	469	321	11068	99	610	105	242	50	410	327	687	5 ITIMs
***Ssa-NILT4***	46	275	354	8112	-	-	204	941	96	212	44	1728	318	1181	4 ITIMs
***Ssa-NILT5***	46	275	342	>2381	-	-	117	619	96	288	50	227	282	443	4 ITIMs
***Ssa-NILT6***	46	275	348	1789	-	-	111	1072	96	308	50	526	294	2230	4 ITIMs
***Omy-NILT1^S^***	88	182	336	1061	318	-	-	-	-	-	-	-	-	162	ITIM/ITAM
***Omy-NILT1^L^***	88	182	336	1061	318	~5800	141	-	69	-	-	-	417	404	ITIM/ITAM
***Omy-NILT2***	46	N.D.	342	N.D.	-	-	132	N.D.	90	N.D.	44	N.D.	135	*391	1 ITAM
***Omy-NILT3***	46	N.D.	330	N.D.	339	N.D.	99	N.D.	105	N.D.	50	N.D.	330	*83	3 ITIMs
***Omy-NILT4^S^***	46	N.D.	330	N.D.	-	N.D.	117	N.D.	105	N.D.	50	N.D.	327	549	4 ITIMs
***Omy-NILT4^L^***	46	N.D.	330	N.D.	321	N.D.	117	N.D.	105	N.D.	50	N.D.	327	549	4 ITIMs
***Dre-NILT1***	40	241	345	197	-	-	117	1243	117	82	35	103	132	214	1 ITIM
***Dre-NILT2***	58	91	342	485	309	362	150	80	96	67	62	82	159	509	1 ITIM
***Cca-NILT1***	40	117	324	279	-	-	117	112	96	381	59	91	288	206	1 ITAM
***Cca-NILT2***	49	169	321	276	-	-	81	2381	105	88	35	110	339	270	4 ITIMs

Number and type of putative signalling motifs in each NILT are indicated.

(* denotes no poly-A signal observed, N.D. denotes not determined)

**Table 3:** Comparison by pairwise alignments (Clustal W) of the NILT Ig domains in Atlantic salmon

	Identity						
*Ssa-*NILT1 Ig1		96	58	40	77	79	85
*Ssa-*NILT2 Ig1	98		59	42	78	81	88
*Ssa-*NILT3 Ig1	69	70		32	63	67	62
*Ssa-*NILT3 Ig2	48	48	52		44	38	44
*Ssa-*NILT4 Ig1	84	84	71	52		91	89
*Ssa-*NILT5 Ig1	89	89	72	50	93		84
*Ssa-*NILT6 Ig1	89	91	69	53	93	90	
	**Similarity**						

The table shows comparison of % amino acid identity and similarity.

Two pseudogenes, *Ssa-NILTp1 *and *Ssa-NILTp2*, were identified in the BAC clone sequence as well as two separate pseudogenes each encoding a single Ig domain. The two pseudogenes have many features in common with *Ssa-NILT1-6*, but *Ssa-NILTp1 *contains a stop codon in the Ig1 domain and exon 5 was absent. *Ssa-NILTp2 *contains an Ig1 domain highly similar to the Ig1 domain of *Ssa-NILT3 *(99%). The initial part of a second Ig domain was also identified. However, immediately after the first cysteine in the Ig2 domain encoding exon, a nucleotide deletion results in a shift of reading frame. In addition, exon 3 encoding the connecting peptide was absent.

All NILTs identified in the BAC clone sequence contain several ITIM signalling motifs in the cytoplasmic region encoded by exon 6. Exons bearing ITIM sequences are always in phase 0, i.e. the intron-exon boundary does not interrupt a codon [32]. In addition, many genes belonging to the immunoglobulin superfamily have exons encoding the Ig domain that are in phase 1 [33], which is also observed in the NILT genes from the Atlantic salmon (Figure 3). Having an ITIM-containing exon in phase 0 implies that the signalling properties of the *Ssa*-NILT receptors are functional and phosphorylation of the tyrosine residues in the ITIM motif leading to inhibition is most likely to occur.

The BAC clone analysis resulted in the identification of several NILTs, which suggests the presence of more than six NILTs in Atlantic salmon. We carried out a study using genomic DNA and primers located in conserved regions of the Ig domain. By using this approach we obtained three novel NILT Ig sequences (*Ssa-NILT7-9*) and 19 singletons at the amino acid level out of 81 clones. This implies that NILT genes in Atlantic salmon are a multigene family of related genes. Interestingly, none of the 81 clones contained the CX_6_C motif, which suggests that a higher number of genes encoding Ig domains containing the CX_3_C motif may be present in the genome. Multiple alignment and phylogenetic analysis divided the CX_3_C sequences into two groups, having different motifs located immediately after the third cysteine (Figure 6). Group 1 molecules contained sequences with a SLYY motif and a histidine and leucine residue at positions 20 and 66, respectively. In contrast, group 2 molecules contained sequences with a (P/R)V(F/L)N motif and a glutamine and isoleucine at positions 20 and 66, respectively. The four residue motifs are located in a loop region between beta-strands C and C', when comparing the amino acid sequence to the sequence of NKp44 [7]. This observation corresponds to the theory that most variation is detected in loop-regions. Future studies will determine if these motifs, as well as the general allelic polymorphism of NILT genes seen in a single individual may contribute to different binding specificities for an as yet unknown ligand. Until now only a few ligands have been identified for non-rearranging receptors with the most investigated being the interaction of classical and non-classical MHC class I molecules to KIRs [34]. NILTs have not been identified in any vertebrates other then teleost to date suggesting that this multigene family of receptors are involved in the control of immune responses in fish only and may represent an ancient type of receptor that has expanded in the fish lineage.

## Conclusions

In conclusion, this report reveals a tightly clustered, multigene family of novel immunoglobulin-like transcripts (NILT) in Atlantic salmon. By screening a highly redundant Atlantic salmon BAC library we have identified and characterised the genomic organisation of six genes encoding NILT receptors. The genes show similar characteristics to NILTs previously identified in rainbow trout [24] having highly conserved cysteines in the Ig domain and several inhibitory signalling motifs in the cytoplasmic region. In a single individual, three additional and NILT sequences were discovered at the genomic DNA level, and allowed division of NILTs into two different groups based on a four residue motif after the third cysteine in the Ig domain. Together with the NILTs from the BAC clone sequence we now know that multiple NILT genes exist in teleost fish and it will be interesting to discover if and how they differ functionally.

## Methods

### Screening the Atlantic salmon CHORI-214 BAC library for *NILT *genes

The Atlantic salmon bacterial artificial chromosome (BAC) library, CHORI-214, was obtained from BACPAC Resources, Children's Hospital Oakland Research Institute (CHORI), Oakland, CA. The average insert size of the library is 188 kbp, representing 18-fold genome coverage [35]. In preparation for screening the library, the filters were prehybridised for 1 h at 65°C in a solution consisting of 5×SSC, 5×Denhardt's solution, and 0.5% SDS. The 40-mer oligonucleotide probe (5' GGAGTGGACAAATGGGGACAAGATAACTACATTAATA3') used for screening the library was labelled at the 5' end with γ-^32^P-ATP using T4 polynucleotide kinase (Invitrogen) in a 10 μl volume reaction and incubated for at least 30 min at 37 C before it was added to the hybridisation solution. The labelling reaction included 1 μl of 10 μM probe, 10 U of T4 polynucleotide kinase, 2 μl of 5× forward reaction buffer, 2 μl of γ-^32^P-ATP (3000 Ci/mmol) and 4 μl of water. The hybridisation solution was as above and to each prehybridised filter 1.6 μl of radioactively labelled oligonucleotide probe were added and the hybridisation was carried out at 65°C overnight (~18 h). Following hybridisation, the filters were washed three times in 1×SSC and 0.1% SDS at 50°C for 1 h for each wash. The washed filters were exposed to phosphor screens overnight and scanned using the Typhoon 9410 Phosphor Imager (Amersham Biosciences). The BAC clones positive by hybridisation were picked from the library and grown in 5 ml of LB medium containing 20 μg/ml chloramphenicol for 14-16 h at 37°C with shaking at 250-300 rpm. PCR amplification of the hybridisation-positive clones was performed in a Thermal Cycler (Biometra) using NILT specific primers (Ss-NILT-f: GCAGAGATCAGATGCCCCTA, Ss-NILT-r: ATCTTGTCCCCATTTGTCCA) to determine if these BACs contain sequences corresponding to the NILT gene. All PCR amplifications were performed in a 25 μl reaction volume using the following conditions: initial denaturation at 95°C for 5 min followed by 35 cycles at 95°C for 45 s, 65°C for 45 s, 72°C for 45 s and finally one cycle at 72°C for 5 min. For each DNA amplification, 0.05 U *Taq *DNA polymerase, 12.5 pmoles of each primer, 1× PCR buffer containing 1.5 mM MgCl_2_, 50 μM of dNTPs and 25 ng of BAC DNA template were used. The amplification products were analysed by electrophoresis through a 1.5% agarose gel containing 1× TBE and 0.5 μg/ml ethidium bromide and visualised using a UV trans-illuminator (Ultra-Violet Products).

### Shotgun sequencing, assembly and annotation of the BAC S0024B13

Isolation of the BAC DNA and shotgun library construction were carried out as described by Johnstone et al. [36]. Briefly, the isolated BAC DNA was sheared by sonication, blunt-end repaired using the End-It™DNA End-Repair Kit (Epicentre) and size fractionated by agarose gel electrophoresis. The region containing 2 to 5 kb fragments was excised and gel purified using a Gel Purification Kit (Qiagen) following the manufacturer's instructions. The fragments were ligated into pUC19 plasmid, which had been cut with *Sma*I and treated with shrimp alkaline phosphatase, and then used to transform *E. coli *XL1 Blue supercompetent cells (Stratagene). Large-scale colony picking was performed using a QPix-2 colony picker (Genetix) and 2,304 clones were sent for sequencing at the Michael Smith Genome Sciences Centre (Vancouver). Base calling and assembling were done using Phred and Phrap [37,38]. Assembled sequences were viewed and edited using Consed [39].

GENSCAN [27] was used to predict novel genes and to identify open reading frames. The predicted gene sequences were analysed for the presence of a leader peptide using SignalP 3.0 [40] and transmembrane regions using the TMHMM server 2.0 http://www.cbs.dtu.dk/services/TMHMM-2.0. Identity as well as similarity with other known sequences was determined using BLAST-n and BLAST-p [28]. Comparisons between sequences were performed with Clustal W [41] and phylogenetic and molecular evolutionary analysis was conducted using MEGA 4 [42].

### Amplification of *NILT *from Atlantic salmon

Genomic DNA was extracted from an Atlantic salmon liver using a genomic DNA purification system (Promega) following the manufacturer's instructions. The isolated genomic DNA was used as template in a PCR with Ss-pan-NILT primers (Table 1).

The reactions were performed in a total volume of 50 μl using 1 U Platinum *Taq *DNA polymerase High Fidelity (Invitrogen) in the presence of 200 nM primer, 250 ng template, 5 μl 10× reaction buffer, 2 mM MgSO_4 _and 200 μM dNTPs. The PCR conditions were: initial denaturation at 94°C for 1 min followed by 25 cycles at 94°C for 15 s, 55°C for 15 s, 68°C for 30 s and finally one cycle at 68°C for 5 min. Atlantic salmon cDNA synthesised from total RNA isolated from head kidney, as previously described [24], was used as template in a PCR with primers designed against NILT. The reactions were performed in a total volume of 50 μl using 1.25 U GoTaq DNA polymerase (Promega) in the presence of 200 nM primer, 300 ng template, 10 μl 5× reaction buffer, 1.5 mM MgCl_2 _and 200 μM dNTPs. The PCR conditions were: initial denaturation at 95°C for 2 min followed by 30 cycles at 95°C for 30 s, 55°C for 30 s, 72°C for 1 min and finally one cycle at 72°C for 7 min. All PCR products were visualised on 1.5% agarose gels containing ethidium bromide and ligated into the pGEM^®^-T Easy vector (Promega) following the manufacturer's instructions. Plasmids were transformed into chemically competent *E. coli *cells, RapidTrans™TAM1 (Active Motif) and recombinant clones identified. Plasmid DNA was purified from overnight cell cultures of recombinant clones using a QIAprep Spin Miniprep Kit (Qiagen) following the manufacturer's instructions. Approximately 1 μg of purified plasmid DNA was used for sequencing with the vector-specific primers T7 and SP6 by MWG-Biotech (Germany).

## Authors' contributions

AEØ carried out the molecular studies, sequence data analysis, annotations and drafted the manuscript. KPL performed the BAC library screening and contig assembly. SAMM, RJMS, WSD and CJS contributed to the planning, design, and direction of the project. All authors read and approved the final manuscript.
